# Adiponectin Effect on The Viability of Human Endometrial Stromal Cells and mRNA Expression of Adiponectin Receptors

**Published:** 2013-03-06

**Authors:** Somayeh Bohlouli, Mozafar Khazaei, Masoud Teshfam, Hosein Hassanpour

**Affiliations:** 1 Department of Veterinary Physiology, Science and Research Branch, Islamic Azad University, Tehran, Iran; 2 Fertility and Infertility Research Center, Kermanshah University of Medical Sciences, Kermanshah, Iran; 3 Department of Basic Sciences (Veterinary Physiology Division), Faculty of Veterinary Medicine, Shahrekord University, Shahrekord, Iran

**Keywords:** Adiponectin, Stromal Cells, Adipo R1, Adipo R2, Endometrium

## Abstract

**Background:**

Adiponectin is one of the most important adipokines secreted from fatty tissue that has a direct inhibitory effect on the development of cancer cells. Adiponectin plays an important role in human reproduction system and fertility of women. Adiponectin concentration decreases in women with endometriosis and endometrial cancer. The aim of the present study was to investigate the effect of adiponectin on human endometrial stromal cell (HESC) viability as well as mRNA expression of Adipo R1 and Adipo R2 receptors.

**Materials and Methods:**

In this experimental study, eight endometrial biopsies were taken and stromal cells were separated by enzymatic digestion and cell filtrations. Stromal cells of each biopsy were divided into four groups: control, 10, 100, and 200 ng/ml adiponectin concentrations. The effect of adiponectin on viability of the normal HESCs was studied by trypan blue staining and the relative expression levels of Adipo R1 and R2 were analyzed by semi-quantitative reverse transcription polymerase chain reaction (RT-PCR). Data were analyzed by one way ANOVA and unpaired student’s t test and p<0.05 was considered significant.

**Results:**

Adiponectin decreased viability of normal human endometrial stromal cells in a dose and time dependent manner. Expression of Adipo R1 and Adipo R2 receptors did not change in the presence of adiponectin.

**Conclusion:**

Adiponectin can directly influence the viability of HESCs and decrease their viability, but it didn’t change expression of adiponectin receptors.

## Introduction

Adiponectin is one of the most important members of adipokine family which is widely synthesized and secreted by fatty tissue. Various roles have been identified for adiponectin such as regulation of glucose level and lipids homeostasis. Furthermore, adiponectin plays a pivotal role in reproductive system (1, 2). Adiponectin is abundantly present in the blood stream and its concentration in human plasma is 5-30 μg/ ml which comprises 0.01% of all proteins in the plasma (3).

Decreasing adiponectin of plasma is indicative of obesity and diabetes (4). Also, various studies have demonstrated that decreasing adiponectin plasma level is linked to increasing the risk of several type of cancer, including breast cancer (5), colorectal (6), prostate (7), and digestive system (8). Adiponectin binds to receptors, known as Adipo R1 and Adipo R2 (9). These receptors contain seven transmembrane domains but differ from G-protein coupled receptors structurally and functionally. The tendency of adiponectin receptors to bind to adiponectin isoforms as well as tissue distribution of these receptors are different (10).

In mice, Adipo R1 exists in different organs such as skeletal muscle, lung, and spleen; whereas Adipo R2 is mainly expressed in liver (11). In human, Adipo R1 and Adipo R2 are expressed in islets of Langerhans, macrophages, adipocytes, and vascular smooth muscles (12- 14).

Various data have indicated that adiponectin is influential in female fertility and plays an important role in female reproductive system. Study has indicated that serum adiponectin level decreases in women with endometriosis (15) and endometrial cancer (16). Also, adiponectin level in peritoneal fluid of endometriosis patients decreased dramatically in advanced endometriosis (17).

In histopathological studies of endometriosis tissues, stromal cells and glands are abundantly present, but changes of endometrial stromal cells are much more than those of endometriosis identifying glands and there is the possibility of the presence of gland-free stromal cells in endometriosis tissue (18). HESCs play pivotal role in female reproductive biology and there is no report on the effect of adiponectin on these cells. The aim of the present study was to examine the effect of adiponectin on human endometrial stromal cells and *in vitro* mRNA expression of adiponectin receptors.

## Materials and Methods

### Samples

In this experimental study, endometrial tissues were taken from women aged 25-35 who had no record of hormonal treatment for three months before surgery and had undergone hysterectomy surgery or biopsy diagnosis for infertility management and reasons other than endometrial malignancies such as myoma. Eight samples of normal endometrium in the secretory phase were taken. The Ethics Committee of Kermanshah University of Medical Sciences and Tehran Science and Research Branch of Islamic Azad University accepted the work on human endometrial tissue in this study and all patients signed informed consents.

### Separation and culture of human endometrial stromal cells

Stromal cells were separated from endometrial tissue according to previous work (19, 20). Each endometrial sample was prepared in sterile condition and was washed with PBS solution containing 1% antibiotic/antimycotic, and then was chopped mechanically. The sample was incubated with collagenase type I solution (2 mg/ml in DMEM/F12) (Sigma, Germany) for 60-90 minutes. The cell suspension was passed through 70 and 40 μm cell strainers (BD falcon, USA) respectively, centrifuged for 15 minutes (2500 rpm) and DMEM/F12 (Gibco, Germany) was added to the cell pellet. Then the suspension was layered on ficoll (Amersham, Sweden) and was centrifuged for 30 minutes (1500 rpm). The stromal cells were collected and were washed with PBS and were cultured in DMEM/ F12 containing 10% fetal bovine serum (FBS) (Gibco, Belgium), 0.1 mg/ml streptomycin, and 100UI/ml penicillin. The cultures were incubated in a humidified atmosphere of 95% air and 5% CO_2_ at 37˚C. After seven days, cell density reached confluency and 1×10^5^ cells were transferred to each well of 24-well culture plate.

### Cell treatment

To add adiponectin (high molecular weight, R&D System Minneapolis, MN USA) to stromal cells, the media was removed and cells were washed with PBS and incubated with serum-free media overnight and then were treated with adiponectin at 0, 10, 100, and 200 ng/ml in 24, 48, and 72 hours for each dose (21).

### Evaluation of cells viability

To analyze the viability of cells, we used trypan blue staining. The stained and non-stained cells were counted by hemocytometer and the percentage of the cells viability was calculated by dividing the number of non-stained cells by total number of cells multiplied by 100 (22).

### Reverse transcription polymerase chain reaction (RT-PCR)

Total RNA was extracted from stromal cells in control group and adiponectin group (100 ng/ml for 48 hours) using RNA purification kit (Jena Bioscience, GmbH, Germany). Total RNA (≥1μg) was used to synthesize complementary DNA (cDNA) in a 20 μl reaction by AccuPower ® RocketScriptTM RT PreMix kit (BIONEER, Korea) and oligo(dT). The PCR was performed using PCR PreMix kit (BIONEER, Korea) according to the manufacturer’s instructions. Cycle conditions were as follows: initial denaturation at 94˚C for 10minutes; followed by 35 cycles of denaturation at 94˚C for 60 seconds, annealing at 58˚C (GAPDH) and 62˚C) Adipo R1 and Adipo R2 (for 60 seconds and extension at 72˚C for 60 seconds, with a final extension at 72˚C for 10 minutes (Table 1). Since less than 35 cycles produced PCR products at low intensity, the PCR reactions were thought to be still in the exponential phase. Experiments were performed in triplicate to ensure reproducibility.

### Semi-quantitative reverse transcription–polymerase chain reaction analysis

The expression of target genes was quantified against the internal reference gene (GAPDH). Products were electrophoresed on a 1.5% agarose gel. Gels were stained with ethidium bromide (10 μg/ mL) and photographed on an ultraviolet transilluminator (UVIdoc; Uvitec, Cambridge, UK). Gel images were analyzed using the UN-SCAN-IT program. Semi-quantitative RT-PCR values were presented as a ratio of the density of Adipo R1 and Adipo R2 bands divided by density of GAPDH bands. RTPCR was performed as three individual replicates.

### Statistical analysis

Data are reported as means ± SEM and statistical analysis was done by SPSS (version 16) using one way analysis of variance (ANOVA) followed by tukey test. The significance of differences in expression of mRNA between two groups was determined using the unpaired Student’s t test. P<0.05 was considered significant.

**Table 1:** Characteristics of the primers used for target genes and internal control


Gene	Primer sequences (5′-3′)	Annealing temperature (˚C)	RT-PCR product size (bp)

**GAPDH**	Forward	CCAGGTGGTCTCCTCTGACTTCAAC	58	224
	Reverse	AGGGTCTCTCTCTTCCTCTTGTGTGCTC		
**Adipo R1**	Forward	AAACTGGCAACATCTGGACC	62	288
	Reverse	GCTGTGGGGAGCAGTAGAAG		
**Adipo R2**	Forward	ACAGGCAACATTTGGACACA	62	300
	Reverse	CCAAGGAACAAAACTTCCCA		


## Results

### Effect of Adiponectin on the viability of human endometrial stromal cells

**Fig 1:**
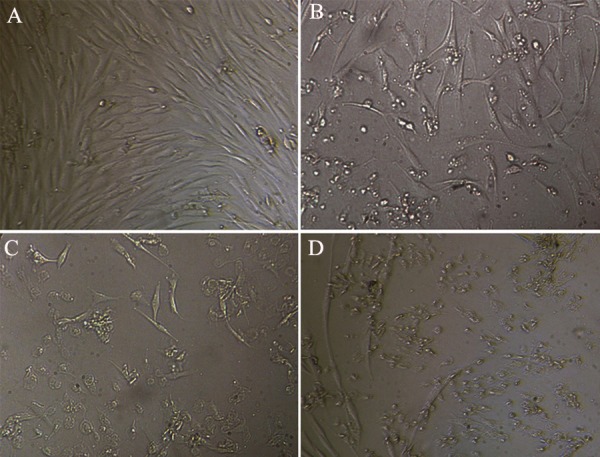
Morphology of normal human endometrial stromal cells in the presence of adiponectin: Control group: (A ×100), 10 ng/ml group: (B ×100), 100 ng/ml group: (C ×100), 200 ng/ml group: (D ×100).

**Fig 2:**
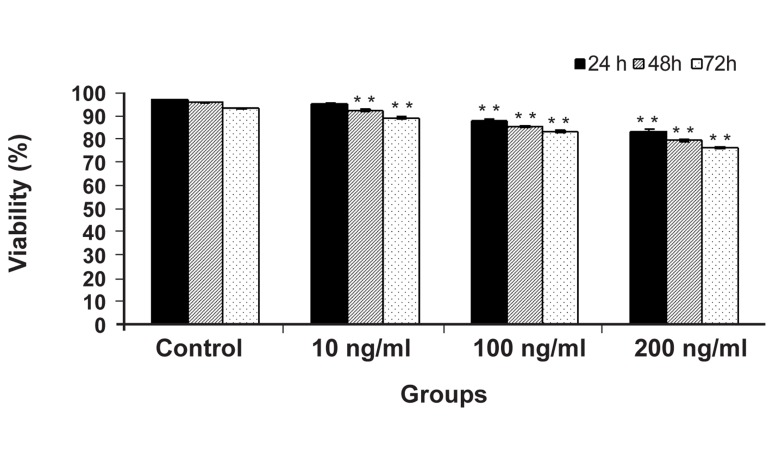
Effect of Adiponectin on viability of normal human endometrial stromal cell. Equal numbers of cells were exposed to adiponectin (10, 100, and 200 ng/ml) for 24, 48, and 72 hours and cells viability was examined by trypan blue staining method. Adiponectin decreased cell viability depending on dose and time and caused cell death. Columns marked with asterisk indicate the significant difference compared to control group (p<0.001).

Human endometrial stromal cells were treated with adiponectin (10. 100, and 200 ng/ml) for 24, 48, and 72 hours. Treatment with adiponectin decreased the viability of stromal cells depending on dose and time (Fig 1). 100 and 200 ng/ml doses in all of the experiment times indicated a significant difference compared to control group, so cell viability in adiponectin (200 ng/ml) was 76.3% after 72 hours (p<0.001) (Fig 2). Adiponectin (10 ng/ ml) showed significant difference only in 48 and 72 hours in comparison with control group (p<0.001).

Equal numbers of cells were incubated for 48 hours with various concentrations of 10 ng/ml (B), 100 ng/ml (C), and 200 ng/ml (D) adiponectin. As it is indicated, treatment with higher concentrations of adiponectin has resulted in a significant decrease in cells viability in comparison with the control group (A).

Equal numbers of cells were exposed to adiponectin (10, 100, and 200 ng/ml) for 24, 48, and 72 hours and cells viability was examined by trypan blue staining method. Adiponectin decreased cell viability depending on dose and time and caused cell death. Columns marked with asterisk indicate the significant difference compared to control group (p<0.001).

### Expression of mRNA AdipoR1 and AdipoR2 in normal human endometrial stromal cells

**Fig 3:**
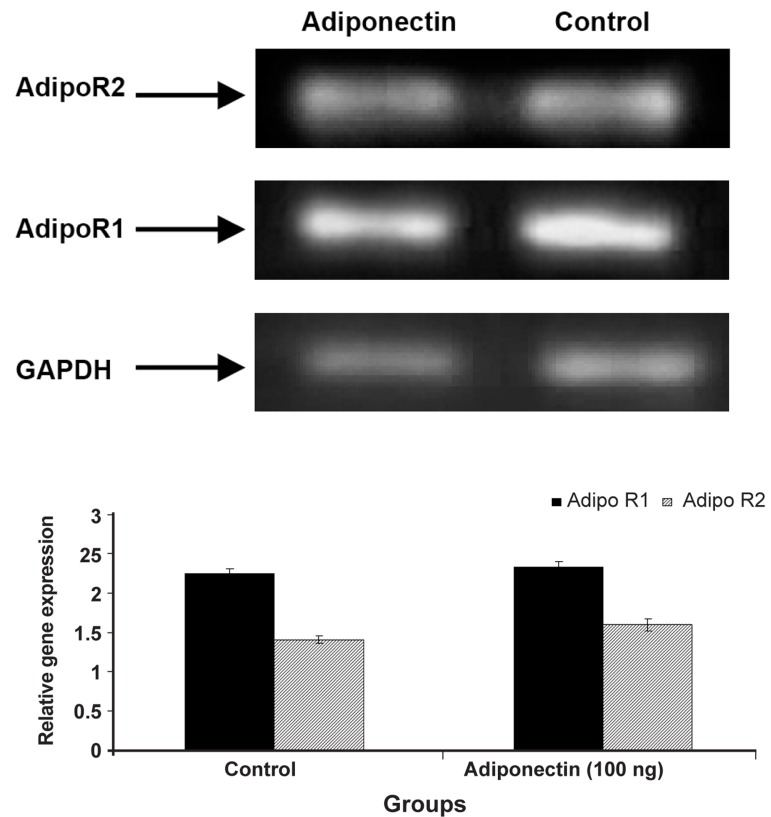
Expression of Adipo R1 and Adipo R2 in normal human endometrial stromal cells with and without Adiponectin in the secretory phase was demonstrated by semi-quantitative RT-PCR analysis. Expression of Adipo R1 and Adipo R2 mRNA in control group and treatment group (100 ng/ml adiponectin) did not indicate significant difference (p>0.05).

In this study, expression of Adipo R1 and Adipo R2 in normal human endometrial stromal cells in the secretory phase in the presence and absence of Adiponectin was demonstrated by semi-quantitative RT-PCR analysis (Fig 3). The results revealed that expression of Adipo R1 and Adipo R2 mRNA in control group (without Adiponectin) and treatment group (100 ng/ml adiponectin) for 48 hours did not indicate significant difference (p>0.05).

## Discussion

In our study, the *in vitro* effect of adiponectin on viability of normal HESCs and expression of Adipo R1 and Adipo R2 receptors was examined. The findings indicated that adiponectin depending on dose and time decreased the viability of HESCs significantly. This finding, confirms the findings reported by Cong et al. which regard to the inhibitory effect of adiponectin on the endometrial carcinoma cell lines (HEC-1-A and RL95-2) in the culture (23) as well as anti-proliferative effects on trophoblast cells and trophoblast cell lines (JEG-3 and BeWO) and decreasing their numbers in the culture (21).

The effects of adiponectin on cell death and decreasing stromal cells count, in this study, was observed with concentrations much lower than normal level which is normally circulating in human blood serum (24, 25). Furthermore, the obtained results in this study are compatible with the findings of previous research regarding the decreasing impact of adiponectin on the viability of various cancer cells such as breast cancer cell line (MCF7), prostate, endothelial cancer and bone cells (26-29).

Lower level of adiponectin is an independent risk factor in the incidence of infertility and reproduction and different genital cancers in epidemiological studies. The direct and indirect mechanisms that influence this phenomenon are not still well-known (30). However, it seems that adiponectin exerts its biological effects through two receptors named Adipo R1 and Adipo R2. Takemura et al. in 2006 showed the expression of two receptors of adiponectin in the epithelial and endometrial stromal cells of endometrial tissue (31). The presence of these two receptors in various normal tissues and cancer cells has been confirmed (32-34).

In the present study, the expression of Adipo R1 and Adipo R2 mRNA in the absence of adiponectin as well as presence of 100 ng/ml adiponectin in normal stromal cells was analyzed. Expression of these two receptors in these cells was observed which confirms the findings of previous studies in which the presence of these receptors in endometrial stromal cells in both secretory and proliferative phases was demonstrated (31). However, the results of our study revealed that adiponectin did not have significant effect on the expression of Adipo R1 and Adipo R2 receptors.

In another study, the expression of Adipo R1 and Adipo R2 receptors in human normal endometrial and endometrial cancerous tissue in the presence of adiponectin (*in vitro*) was investigated. The findings showed that adiponectin decreased cell proliferation in human endometrial cancerous tissue via adiponectin receptors and the level of Adipo R1 expression was higher than that of Adipo R2 but the level of expression of receptors in cancerous tissue did not indicate significant difference compared to normal non-cancerous tissue (30). The recent research has indicated that expression of Adipo R1 in breast cancer cells (32) and human endometrial cancerous tissue (23) is higher than th at of Adipo R2.

These findings are compatible with the results of our study. The reason for this could be because of adiponectin binding to Adipo R1 and Adipo R2 receptors and the ability of these receptors in activating ligand-dependent AMP-activated protein kinase (AMPK). Activation of AMPK results in decreasing cell proliferation and increasing the number of inhibited cells in G_1_/G_0_ phase and consequently inducing cell death (35).

## Conclusion

Adiponectin inhibit endometrial stromal cell proliferation in dose and time dependant manner, and cause cell death. It can suggest as anti-endometriosis agent.

For further studies on the effect of adiponectin in inhibition of progressive development and proliferation of endometriotic cells, endometrial stromal cells of endometriosis patients should be used and the function and expression of its receptors in the development of the disease must be investigated.

## References

[1] Garaulet M, Hernández-Morante JJ, de Heredia FP, Tébar FJ (2007). Adiponectin, the controversial hormone. Public Health Nutr.

[2] Zavalza-Gómez AB, Anaya-Prado R, Rincón-Sánchez AR, Mora-Martínez JM (2008). Adipokines and insulin resistance
during pregnancy. Diabetes Res Clin Pract.

[3] Wang Y, Lam KS, Yau MH, Xu A (2008). Post-translational modifications
of adiponectin: mechanisms and functional implications. Biochem J.

[4] Engeli S, Feldpausch M, Gorzelniak K, Hartwig F, Heintze U, Janke J (2003). Association between adiponectin and
mediators of inflammation in obese women. Diabetes.

[5] Chen DC, Chung YF, Yeh YT, Chaung HC, Kuo FC, Fu OY (2006). Serum adiponectin and leptin levels in Taiwanese
breast cancer patients. Cancer Lett.

[6] Otake S, Takeda H, Suzuki Y, Fukui T, Watanabe S, Ishihama K (2005). Association of visceral fat accumulation
and plasma adiponectin with colorectal adenoma: evidence
for participation of insulin resistance. Clin Cancer Res.

[7] Freedland SJ, Sokoll LJ, Platz EA, Mangold LA, Bruzek DJ, Mohr P (2005). Association between serum adiponectin,
and pathological stage and grade in men undergoing
radical prostatectomy. J Urol.

[8] Ishikawa M, Kitayama J, Kazama S, Hiramatsu T, Hatano K, Nagawa H (2005). Plasma adiponectin and gastric cancer. Clin Cancer Res.

[9] Yamauchi T, Nio Y, Maki T, Kobayashi M, Takazawa T, Iwabu M (2007). Targeted disruption of Adipo R1 and
Adipo R2 causes abrogation of adiponectin binding and
metabolic actions. Nat Med.

[10] Kadowaki T, Yamauchi T (2005). Adiponectin and adiponectin receptors. Endocr Rev.

[11] Yamauchi T, Kamon J, Ito Y, Tsuchida A, Yokomizo T, Kita S (2003). Cloning of adiponectin receptors that mediate
antidiabetic metabolic effects. Nature.

[12] Kharroubi I, Rasschaert J, Eizirik DL, Cnop M (2003). Expression
of adiponectin receptors in pancreatic beta cells. Biochem
Biophys Res Commun.

[13] Chinetti G, Zawadski C, Fruchart JC, Staels B (2004). Expression
of adiponectin receptors in human macrophages and regulation
by agonists of the nuclear receptors PPAR alpha,
PPAR gamma, and LXR. Biochem Biophys Res Commun.

[14] Arita Y, Kihara S, Ouchi N, Takahashi M, Maeda K, Miyagawa J (1999). Paradoxical decrease of an adipose-specific
protein, adiponectin, in obesity. Biochem Biophys Res Commun.

[15] Takemura Y, Osuga Y, Harada M, Hirata T, Koga K, Morimoto C (2005). Serum adiponectin concentrations are
decreased in women with endometriosis. Hum Reprod.

[16] Soliman PT, Wu D, Tortolero-Luna G, Schmeler KM, Slomovitz BM, Bray MS (2006). Association between
adiponectin, insulin resistance, and endometrial cancer. Cancer.

[17] Yi KW, Shin JH, Park HT, Kim T, Kim SH, Hur JY (2010). Resistin
concentration is increased in the peritoneal fluid
of women with endometriosis. Am J Reprod Immunol.

[18] Takayama K, Zeitoun K, Gunby RT, Sasano H, Carr BR, Bulun SE (1998). Treatment of severe postmenopausal endometriosis
with an aromatase inhibitor. Fertil Steril.

[19] Khazaei M, Chobsaz F, Khazaei S (2010). The effect of different
doses of clomiphene citrate on morphology and proliferation
of human endometrial stromal cells in in-vitro
culture. Babol J Med Sci.

[20] Esfandiari N, Ai J, Khazaei M, Nazemian Z, Jolly A, Casper RF (2008). Angiogenesis following three-dimensional
culture of isolated human endometrial stromal cells. Int J
Fertil Steril.

[21] Benaitreau D, Dieudonné MN, Dos Santos E, Leneveu MC, Mazancourt Pd, Pecquery R (2009). Antiproliferative effects
of adiponectin on human trophoblastic cell lines
JEG-3 and BeWo. Biol Reprod.

[22] Freshney R (2005). Culture of animal cells: A manual of basic
technique.

[23] Cong L, Gasser J, Zhao J, Yang B, Li F, Zhao AZ (2007). Human
adiponectin inhibits cell growth and induces apoptosis in
human endometrial carcinoma cells, HEC-1-A and RL95-2. Endocr Relat Cancer.

[24] Corbetta S, Bulfamante G, Cortelazzi D, Barresi V, Cetin I, Mantovani G (2005). Adiponectin expression in human
fetal tissues during mid- and late gestation. J Clin Endocrinol
Metab.

[25] Kajantie E, Hytinantti T, Hovi P, Andersson S (2004). Cord plasma
adiponectin: a 20-fold rise between 24 weeks gestation
and term. J Clin Endocrinol Metab.

[26] Dieudonne MN, Bussiere M, Dos Santos E, Leneveu MC, Giudicelli Y, Pecquery R (2006). Adiponectin mediates antiproliferative
and apoptotic responses in human MCF7
breast cancer cells. Biochem Biophys Res Commun.

[27] Bråkenhielm E, Veitonmäki N, Cao R, Kihara S, Matsuzawa Y, Zhivotovsky B (2004). Adiponectin-induced antiangiogenesis
and antitumor activity involve caspasemediated
endothelial cell apoptosis. Proc Natl Acad Sci
USA.

[28] Berner HS, Lyngstadaas SP, Spahr A, Monjo M, Thommesen L, Drevon CA (2004). Adiponectin and its
receptors are expressed in bone-forming cells. Bone.

[29] Bub JD, Miyazaki T, Iwamoto Y (2006). Adiponectin as a growth
inhibitor in prostate cancer cells. Biochem Biophys Res
Commun.

[30] Moon HS, Chamberland JP, Aronis K, Tseleni-Balafouta S, Mantzoros CS (2011). Direct role of adiponectin and adiponectin
receptors in endometrial cancer: in vitro and ex
vivo studies inhumans. Mol Cancer Ther.

[31] Takemura Y, Osuga Y, Yamauchi T, Kobayashi M, Harada M, Hirata T (2006). Expression of adiponectin receptors
and its possible implication in the human endometrium. Endocrinology.

[32] Körner A, Pazaitou-Panayiotou K, Kelesidis T, Kelesidis I, Williams CJ, Kaprara A (2007). Total and high-molecularweight
adiponectin in breast cancer: in vitro and in vivo
studies. J Clin Endocrinol Metab.

[33] Motoshima H, Wu X, Mahadev K, Goldstein BJ (2004). Adiponectin
suppresses proliferation and superoxide generation
and enhances eNOS activity in endothelial cells
treated with oxidized LDL. Biochem Biophys Res Commun.

[34] Luo XH, Guo LJ, Yuan LQ, Xie H, Zhou HD, Wu XP (2005). Adiponectin stimulates human osteoblasts proliferation
and differentiation via the MAPK signaling pathway. Exp Cell Res.

[35] Reynolds RK, Hu C, Baker VV (1998). Transforming growth
factor-alpha and insulin-like growth factor-I, but not epidermal
growth factor, elicit autocrine stimulation of mitogenesis
in endometrial cancer cell lines. Gynecol Oncol.

